# Patient Experiences after Physical Trauma: The Negative Effect of the COVID-19 Pandemic on Recovery

**DOI:** 10.3390/ijerph191912258

**Published:** 2022-09-27

**Authors:** Jeanette Finstad, Thomas Clausen, Leiv Arne Rosseland, Olav Røise, Ingrid A. Havnes

**Affiliations:** 1 Division of Emergencies and Critical Care, Department of Research and Development, Oslo University Hospital, P.O. Box 4956, 0424 Oslo, Norway; 2 Institute of Clinical Medicine, University of Oslo, P.O. Box 1171, 0318 Oslo, Norway; 3 Norwegian Centre for Addiction Research, Institute of Clinical Medicine, University of Oslo, P.O. Box 1039, 0315 Oslo, Norway; 4 Norwegian Trauma Registry, Division of Orthopedic Surgery, Oslo University Hospital, P.O. Box 4956, 0424 Oslo, Norway; 5 Division of Mental Health and Addiction, Oslo University Hospital, P.O. Box 4959, 0424 Oslo, Norway

**Keywords:** COVID-19, pandemic, physical trauma, recovery, rehabilitation, mental health, substance use, qualitative research

## Abstract

The coronavirus disease 2019 (COVID-19) pandemic generated a crisis within the healthcare system, during which acute, COVID-19-related health needs were prioritized over less urgent needs, including vulnerable subgroups. This study explored experiences of recovery among survivors of physical injuries associated with severe pain during the COVID-19 pandemic in Norway. In-depth interviews were conducted among 13 participants. Findings generated by a thematic analysis revealed that the pandemic, including the contagion control measures and interrupted healthcare, were of negative consequence for the participants’ recovery experiences and mental and physical health. Despite experiencing severe pain and perceived needs for support, the participants experienced being deprioritized by the healthcare system. They experienced a reduced capacity to cope with pandemic-related stress and to perform everyday tasks, which they perceived as generating an additional burden for their loved ones. Alcohol was reported to be used in an effort to relieve the associated mental distress. As suggested by this study, injury survivors constitute a vulnerable subgroup for whom the continuity of rehabilitation services during a national crisis, as well as the integration of mental health support, can be essential for mitigating the negative impact of the crisis on recovery and for promoting optimal long-term health outcomes.

## 1. Introduction

The coronavirus disease 2019 (COVID-19) pandemic, which remains of significant public health concern worldwide [1], reached Norway in February 2020. Consequently, the government enforced restrictive contagion control measures, including a national lockdown, mandatory isolation, and quarantine following infection and exposure, and social and physical distancing requirements [2,3]. While these measures were essential for controlling the spread of COVID-19, studies have shown that they also impacted the general population’s mental health [4]. In addition, the healthcare system faced significant, acute challenges when faced with a surge of COVID-19-related hospital admissions, which reduced less critical public health services, such as outpatient consultations and rehabilitation services [5]. The economic and social consequences of COVID-19, including interrupted healthcare services, affected subgroups of vulnerable patients, such as individuals with pre-existing mental illness, chronic pain, and substance use disorders, making them prone to adverse health outcomes [5,6,7].

Physical injury is the leading cause of death globally, and it is estimated that 10% of all the years living with a disability is associated with people who suffer nonfatal injuries [8]. Studies have shown that survivors of physical trauma often experience reduced physical function, including pain, and are at risk of developing opioid use disorders and mental health challenges [9,10]. Additionally, many trauma survivors require long-term rehabilitation support and follow-up. These factors distinguish survivors of physical trauma as a vulnerable subgroup for whom a crisis, such as the COVID-19 pandemic, risks adverse health outcomes.

This article, based on an ongoing study exploring experiences of long-term recovery among survivors of physical trauma [9], investigates such experiences during the COVID-19 pandemic and the impact on recovery. Additionally, this article reflects on the methodological and ethical challenges associated with conducting qualitative research during a pandemic.

## 2. Methods

The findings presented in this paper are derived from the data produced by a qualitative follow-up study of physical trauma survivors’ experiences of trauma care and recovery 18 months after injury. These longitudinal data build on a study that explored the participants’ post-trauma experiences six weeks after discharge from the trauma center [9]. The COREQ checklist was used when planning and reporting this qualitative study [11].

### 2.1. Setting and Recruitment

The participants in this study were recruited from Oslo University Hospital (OUH), a level 1 trauma center [12], with a patient population of 3.1 million, constituting 56% of the 5.5 million inhabitants of Norway. Together with three other trauma centers and 35 hospitals, OUH forms a national trauma system that is dedicated to receiving and treating severely injured patients in Norway [13,14].

### 2.2. Data Collection

Participants over 18 years old, able to consent, and with a complex and/or severe surgical trauma, including orthopedic injuries associated with severe pain, were included [9]. The participants were recruited with purposive sampling and identified at the trauma centers’ daily meetings. First, the ward nurse asked the patients whether the researcher could meet them and provide information about the study. Then the researcher (J.F.) provided oral and written information about the study. Of the 13 men and 3 women who were asked to participate during the inclusion phase, 11 men and 2 women were included and provided written informed consent. The participants agreed to be contacted by telephone after discharge from the trauma center. After the first interview was conducted six weeks post-discharge, all 13 participants were contacted and agreed to participate in the follow-up interview during the COVID-19 pandemic, 18 months post-discharge. The semi-structured interviews were conducted by the first author (J.F.).

### 2.3. Sample

The study sample comprised 11 men and 2 women with a mean age of 50 years (median = 50, range = 19–78). The following sources of injury were represented: motor vehicle accidents (5), falls (1), hit by a falling object (2), skiing (3), and sled ride accidents (1). All but two had injuries that required surgery, and seven of the 13 participants were transferred from the local hospital to OUH shortly after the accidental trauma, reflecting the severity of the injuries and the study participants’ need for advanced trauma care.

At the time of the physical injury, ten were employed full-time or engaged in full-time study, one worked less than 50%, and two were retired. At the time of the interviews, during COVID-19, three worked full-time or engaged in full-time study, five worked less than 50%, two were on 100% sick leave, and four were retired or on disability benefits.

### 2.4. Analysis

From a longitudinal perspective, to understand the participants’ experiences of recovery across time, including essential turning points [15], the following themes were explored: experiences of pain, use of opioids, post-discharge follow-up, mental health, and quality of life. As the interviews were conducted during the COVID-19 pandemic, the pandemic and its influence on the participants’ everyday lives became a natural part of the dialog and probing questions. For example, when a participant was on sick leave with trauma-related pain and mental health problems and was concurrently responsible for home-schooling children, the pandemic and its impact became an integrated part of the experiences after trauma.

The interviews were recorded and transcribed verbatim before they were processed and coded in NVIVO [16]. For this paper, all data from the original study’s transcript that contained the terms “COVID-19”, “Corona”, and “Pandemic” were extracted using a text search query [17], which lists all sources containing specific words, and thematically analyzed [18] by J.F. and I.A.H., separately. After the initial analysis, J.F. and I.A.H. met regularly to discuss and establish consensus regarding emerging themes. All authors contributed, by means of an interdisciplinary approach [9], to the final stage of analysis, resulting in three themes: Feeling deprioritized by the healthcare system, an experience of reduced capacity to handle stressful tasks, and alcohol consumption to relieve additional mental distress during the pandemic. Each participant is given a pseudonym.

### 2.5. Collecting Data during the COVID-19 Pandemic

Data were collected during September, October, and November 2020, approximately 18 months after the accident. During this period, there was a sharp increase in reported COVID-19 cases and hospitalizations, followed by consistently high infection rates, with a predominance of new cases in the Oslo area. Vaccines were not yet available, and the government recommended reducing social contact, maintaining physical distance, and wearing face masks in public. All participants were contacted by SMS the day before the interview and asked if they had symptoms of COVID-19 and whether they felt comfortable conducting the interview under the circumstances, which were compliant with contagion measures, which included physical distancing and the use of hand sanitizer. As a result, ten interviews were undertaken at the participants’ residences, and three were conducted by telephone due to: potential exposure to COVID-19, fear of infection, and a reduced number of social contacts per governmental regulations.

## 3. Results

All the participants had suffered major orthopedic injuries and described the 18 months between being discharged from the trauma center and participating in the follow-up study as complex and challenging. The recovery pathway had, for several participants, been unpredictable due to unexpectedly unstable periods during which they experienced increased pain, reduced physical function, and affected mental health. The participants also experienced the COVID-19 pandemic as an additional burden that negatively influenced their treatment services and recovery. Specifically, they described feeling deprioritized by the healthcare system and experiencing a reduced capacity to handle stressful tasks. Alcohol use was also reported to relieve the additional mental distress triggered by the pandemic.

### 3.1. Feeling Deprioritized by the Healthcare System

When the national lockdown commenced in March 2020, several participants experienced postponement of planned follow-up services with trauma specialists, and telehealth and digital care were not offered. Among three participants, Helge, Andrine, and Fred, the lack of follow-up resulted in feelings of being deprioritized during the lockdown.

Andrine experienced panic attacks after her initial admission to the trauma center and developed symptoms of posttraumatic stress disorder after discharge. She suffered physical discomfort and was awaiting follow-up surgery. During her recovery, Andrine sought but did not receive professional mental health support, which she explained made her feel rejected and overlooked. She distanced herself from the healthcare system and was left with the impression that her mental and physical health problems did not fulfill the criteria to be prioritized during COVID-19. As she explained:

“I have not heard anything from them, so I will probably call when everyday life starts to calm down a little and ask… because Corona came, so I was unsure if they had other things to do.”

Helges’ trauma occurred a year before lockdown, and, despite suffering severe and increasing pain, he received less attention from the health system than he felt he needed during the COVID-19 pandemic. Although he had been receiving continuous trauma care, including rehabilitation treatment, for several months following his injury, outpatient services became unavailable to him during the pandemic. He felt that his recovery became more difficult due to the postponement of his follow-up care, which he attributed to the prioritization of patients with COVID-19-related health issues:

“The first time I was there, he [the physician] said we must do it [a follow-up clinical examination of the injury]. Then, all that Corona stuff started in March, and then, when summer came, I looked into it. Naturally, they probably had not forgotten about it… it was probably not that easy to arrange.”

Fred’s experience exemplifies how some participants self-assessed their health needs as less likely to be prioritized during the lockdown. Throughout his initial hospital admission, Fred suspected that his hallucinations, paranoia, and severe nightmares were related to the opioids prescribed for his pain. He interpreted his symptoms as indicative of having acquired a severe mental illness [9]. He experienced these symptoms as traumatic and, although they did not continue after discharge, he remained preoccupied with concerns about having developed a severe mental illness. He hoped that these concerns would diminish with time, and thus did not seek support during the first period of his recovery. Nevertheless, 18 months into his recovery, these symptoms still affected his everyday life, and he perceived himself as needing professional help. When the lockdown commenced in March 2020, Fred assessed his mental health challenges as unlikely to be prioritized and delayed contacting the trauma center to discuss whether the symptoms he had experienced during hospitalization could be related to opioid use:

“I must do it [make an appointment] … but I might as well wait a little while now, because now it is corona, which will continue until next year.”

### 3.2. Alcohol to Relieve Additional Mental Distress during the Pandemic

Some participants assessed their physical and mental health at the time of this follow-up study as having worsened in the time since discharge from the trauma center. Terje, for example, experienced few health problems six weeks post-discharge but developed increased and widespread pain several months later, which he interpreted as injury-related, and which reduced his physical function and ability to work. Terje assessed his state of recovery during the early phases of the pandemic and lockdown as unstable. He described experiencing mental distress, including symptoms of anxiety and depression, which he related to his compromised physical health, sick leave, and limited ability to perform and participate in daily activities. To cope, he started drinking alcohol:

“I wouldn’t say it was the physical pain that triggered it… I think it had to do with the mental pain, which was definitely a result of the injury. I did not drink alcohol because I was in pain; I used alcohol because of the mental… fear, guilt, and those kinds of things… but there were several factors. There was corona, there was a job situation that was not optimal… and so I drank too much alcohol.”

Terje explained that his alcohol consumption during the lockdown gradually progressed from one day each week to nearly every day. He described perceiving alcohol as his only way to relieve the mental distress associated with the injury and the pandemic. When his wife expressed concern, he contacted his general practitioner, who referred him to a psychologist.

### 3.3. Reduced Capacity to Handle Stressful Tasks

Ben experienced the lockdown as negatively affecting his well-being and everyday life and reducing his capacity to handle stressful tasks. Ben also had postoperative infections, which required treatment at the hospital, and which his wife was instrumental in facilitating. During the lockdown, Ben was on sick leave and used opioids prescribed for his pain. He experienced that his reduced physical function, including pain, limited his ability to homeschool his children and perform everyday tasks. Consequently, his wife also assumed the predominant share of the responsibility at home, which Ben perceived to be a significant burden on her:

“It has been a completely different everyday life for her after the accident. She was pregnant, and she had two kids she cares for pretty much all of the time, and I have been in and out of hospital… and when Corona came… My last infection was in the middle of the outbreak, so then there was one kid who we were homeschooling and another child who could no longer go to daycare… Yes… It has been challenging.”

## 4. Discussion

The COVID-19 pandemic led to an unexpected crisis within the healthcare system, during which acute, COVID-19-related care needs took precedence over rehabilitation and ongoing or follow-up healthcare services among patients with long-term needs or chronic conditions. The participants of this qualitative study experienced the pandemic as an additional burden during the challenging recovery period. For some, the impact of the lockdown on their everyday lives, which included higher levels of stress and, for some, a reduced capacity to cope with stress, as well as the interruptions to rehabilitation and follow-up care that it entailed, compounded and exacerbated both the physical and mental health burden of recovery. Despite experiencing pain, awaiting follow-up surgery, or desiring recovery support and information about their recovery progression and status, they experienced being deprioritized by the healthcare system. They avoided seeking support and they perceived themselves as needing to prevent being an additional burden on the healthcare system. Several experienced reduced physical and mental health issues, and one developed an unhealthy relationship with alcohol.

During a national health crisis, a sudden and unexpected upsurge of acute health needs may result in the deprioritization of less urgent health needs, and extensive attention, on the part of both the media and the government, may be directed towards the overburdened healthcare system. The participants both experienced and believed that their needs could not be prioritized, which resulted in canceled or delayed follow-up care, unmet health needs, and an experience of decreased physical and psychological well-being. This is of great concern, as disrupted rehabilitation services may compromise the recovery process, leading to cumulative, adverse effects, such as reduced functional outcomes and, subsequently, increased support needs [19].

In the early phase of the pandemic in Norway, there was a rapid adoption of telehealth, including video consultations, particularly in primary healthcare and mental healthcare services [20,21]. The participants did not experience that canceled and postponed appointments from the somatic specialists healthcare services were replaced with telehealth options. However, the specialists considered telehealth as an inappropriate tool for follow-up appointments due to the need for physical examinations, reflecting a sole focus on patients’ physical health needs. Moreover, during the first year of the pandemic, when data were collected, it is likely that the hospital management prioritized resources for emergency care and hospitalized patients [22].

Acute injury survivors are at an increased risk of mental health problems [23], and several participants in the current study reported symptoms of decreased mental health during their recovery period and worsened mental health during the period of recovery that intersected with the COVID-19 pandemic. This finding aligns with the results generated by a meta-analysis that concluded that people with pre-existing mental illnesses are especially vulnerable during a crisis [7]. For example, mental illness may reduce one’s capacity to manage stressors [24]. Certain stressors may trigger symptoms associated with one’s mental illness and may exacerbate the frequency and/or intensity of these symptoms. Moreover, people with mental illness often experience marginalization, and marginalized populations are also at an increased risk during crises [25]. Hence, acute injury survivors may be especially vulnerable to the negative health impacts of a crisis, and this may increase the risk of adverse health outcomes in the long run, thus, underscoring the importance of mapping and meeting their physical and mental health needs during treatment and after discharge from the trauma center with an appreciation of potentially intersecting and interacting vulnerabilities.

There is evidence that the overall trend of alcohol consumption in the general population decreased during the first phase of the pandemic in most European countries [26,27,28] as well as in the USA [29]. However, the minority in studies from Norway and Finland, who increased their alcohol consumption during the pandemic, were more likely to have used alcohol the last year, have a high educational level [28] and live with children [27].

It is well described that stressful events in life, such as unemployment and social isolation, may trigger substance use, including alcohol use as a coping mechanism [30]. Additionally, drinking to cope with negative emotions is associated with a risk of developing alcohol-related problems [31]. During the pandemic, high stress and anxiety levels were significantly associated with increased alcohol use [32]. From a short-term perspective, individuals may temporarily use alcohol to relieve stress and emotional and psychological distress. Still, excessive alcohol intake may lead to an alcohol use disorder and increased mental and physical health problems [33]. There is a knowledge gap regarding trends in alcohol consumption among physical trauma survivors during the pandemic. However, the phenomenon described in this paper, where alcohol is used as a coping strategy when experiencing complex challenges related to both physical injury and the pandemic, may involve a risk of developing a harmful change in alcohol consumption patterns.

Studies conducted prior to the pandemic have shown that an injury survivor’s next of kin suffer a variety of substantial burdens, such as anxiety, depression, and reduced quality of life [34]. In our study, the participants experienced reduced capacity to participate in daily activities and perform the tasks associated with their familial roles during the lockdown, which was perceived as further burdening their next of kin. Moreover, research has demonstrated that people who support people who use alcohol excessively can experience this as a heavy burden, resulting, at times, in occupational dysfunction [35]. This is of great concern, as there is evidence that caregivers who experienced an extensive care burden during the pandemic reported significant impacts on their health, including symptoms of depression and deteriorated well-being [36]. These findings underscore that caregivers can also be understood as a vulnerable subgroup at risk of adverse health outcomes during times of crisis; subsequently, they may need support from the healthcare system during various phases of the crisis.

The current study has several strengths. Firstly, the data were collected during the pandemic, reducing recall bias risk. Secondly, all initial study participants participated in the follow-up study, thus, providing longitudinal data that offers unique insights into the recovery process and progression over time. Thirdly, as this study was not initially designed to focus specifically on COVID-19, the interview guide did not contain pandemic-related questions, and the themes related to the impact of the pandemic on recovery experiences emerged and evolved inductively, which can be understood as strengthening the validity of the study [37]. The study limitations include the low number of participants and their recruitment from a single trauma center. Nevertheless, as this trauma center is Norway’s largest and presumably best resourced facility, it is possible that the negative impact of the pandemic on recovery experiences was even more pronounced among injury survivors in less resource-rich areas. There is also reason to believe that the healthcare system under-reports challenges related to meeting the care needs of its patients. Although the male gender is over-represented in the physical trauma population, it should be noted that only two female participants were included in the study. Hence, gender experiences of recovery could not be explored in depth. This is a limitation as it has been shown that women are at higher risk than men for the worse outcome of functional and psychosocial character after major physical trauma [38], which, in turn, may be important for recovery during COVID-19. Nevertheless, the findings presented in this article cannot be generalized in a statistical sense. Instead, it should be recognized as a contribution to a more nuanced understanding of trauma patients’ experiences with recovery during COVID-19.

Most of the interviews in this study were conducted in person during the pandemic, which required ethical considerations regarding the risk of potential harm [39], such as the possibility of infecting the participants with COVID-19. Furthermore, as the interviews were conducted during a time of government-imposed physical distancing and reduced social contacts (a maximum of ten social contacts was advised), the researcher became one of the few people the participants met. All the interviews could have been conducted by phone but without the benefit of observing nonverbal communication, such as facial expressions, eye contact, and body language. Such nonverbal communication can be important for enabling the interviewer, who was, in this case, an experienced clinician, to assess possible harm and adjust the course of the interview in ways that avoided harm, such as when discussing sensitive topics, including long-term or nonprescribed opioid use, mental health problems, and experiences of loss [40].

## 5. Conclusions

This study has produced knowledge of importance for future crises, including pandemics, by generating insight into possible negative consequences of the redistribution of health resources and deprioritization of vulnerable subgroups, including trauma survivors. The participants in this study were physical trauma survivors, and the findings suggest that continuity of rehabilitation services, including digital outpatient services, and the integration of mental health support can be essential for optimal health outcomes. Moreover, the healthcare system must recognize the potentially harmful implications of a crisis, including any public health measures adopted to address it for patients in long-term recovery, and relevant counteractions, such as telehealth and digital care, should be implemented. Importantly, for this to be feasible, the health system must be sufficiently resourced, which includes an adequate, precrisis state of preparedness.

Overlooked and untreated mental health problems among trauma survivors are a significant burden during recovery, and more attention to mental health during the acute phase of the injury is needed. As suggested by this study, the healthcare system should incorporate follow-up routines for trauma patients with chronic pain and pre- and comorbid mental health and/or substance use problems. By providing tailored treatment to meet trauma survivors’ biopsychosocial health needs, the health system may empower this vulnerable subgroup of patients to cope with the additional burdens of a crisis.

## Data Availability

Not applicable.
